# Development of a deep residual learning algorithm to screen for glaucoma from fundus photography

**DOI:** 10.1038/s41598-018-33013-w

**Published:** 2018-10-02

**Authors:** Naoto Shibata, Masaki Tanito, Keita Mitsuhashi, Yuri Fujino, Masato Matsuura, Hiroshi Murata, Ryo Asaoka

**Affiliations:** 1 Queue inc, Tokyo, Japan; 2 Division of Ophthalmology, Matsue Red Cross Hospital, Shimane, Japan; 3 Department of Ophthalmology, Shimane University Faculty of Medicine, Shimane, Japan; 4 Department of Ophthalmology, The University of Tokyo, Tokyo, Japan; 5 Department of Ophthalmology, Graduate School of Medical Science, Kitasato University, Sagamihara Kanagawa, Japan

## Abstract

The Purpose of the study was to develop a deep residual learning algorithm to screen for glaucoma from fundus photography and measure its diagnostic performance compared to Residents in Ophthalmology. A training dataset consisted of 1,364 color fundus photographs with glaucomatous indications and 1,768 color fundus photographs without glaucomatous features. A testing dataset consisted of 60 eyes of 60 glaucoma patients and 50 eyes of 50 normal subjects. Using the training dataset, a deep learning algorithm known as Deep Residual Learning for Image Recognition (ResNet) was developed to discriminate glaucoma, and its diagnostic accuracy was validated in the testing dataset, using the area under the receiver operating characteristic curve (AROC). The Deep Residual Learning for Image Recognition was constructed using the training dataset and validated using the testing dataset. The presence of glaucoma in the testing dataset was also confirmed by three Residents in Ophthalmology. The deep learning algorithm achieved significantly higher diagnostic performance compared to Residents in Ophthalmology; with ResNet, the AROC from all testing data was 96.5 (95% confidence interval [CI]: 93.5 to 99.6)% while the AROCs obtained by the three Residents were between 72.6% and 91.2%.

## Introduction

Bilateral blindness was estimated to be present in 9.4 million people with glaucoma in 2010, and this number is expected to rise to 11.2 million people in 2020^1^. Glaucoma is an irreversible disease and the second most common cause of blindness worldwide^1^. Early diagnosis of glaucoma is hugely important for preventing blindness. In glaucoma, morphological changes at the optic disc occur in typical patterns^2^. Evaluation of the optic nerve head (ONH) and retinal nerve fiber layer (RNFL) around the optic disc is very important for accurate and early diagnosis of glaucoma since structural changes may precede measurable visual field (VF) loss^3^. With the development of imaging devices, such as optical coherence tomography (OCT)^4^, the Heidelberg Retina Tomograph (HRT, Heidelberg Engineering GmbH, Heidelberg, Germany) and scanning laser polarimetry (GDx: Carl Zeiss Meditec, Dublin, CA), it is possible to measure glaucomatous structural changes quantitatively and in great detail. However, a considerable limitation of these ‘high-tech’ imaging devices is that they are usually available only at specialist eye clinics or hospitals; consequently, glaucoma sufferers – who have not visited these facilities – can persist without a diagnosis for many years. Furthermore, these imaging devices are usually unavailable in poorer nations.

The two-dimensional fundus photograph is a basic ophthalmological screening tool. In Japan and many other countries, the screening of ophthalmological diseases, including glaucoma, is based on expert interpretation of two-dimensional fundus photographs, and more high-tech imaging devices are generally not used. Thus, two-dimensional fundus photography remains a key instrument to prevent blindness through early detection of glaucoma. One of the problems with such fundus photography is that diagnosis is currently based on subjective judgement. Nonetheless, optic disc morphologic information quantified from fundus photographs are highly correlated with structural measurements obtained with HRT and GDx^5^.

The development of deep learning methods represents a revolutionary advance in imaging recognition research^6^. Deep learning methods are similar to artificial neural networks, which process information via interconnected neurons, however, deep learning methods have many ‘hidden layers’ which become computable in conjunction with a feature extractor. The feature extractor transforms raw data into a suitable feature vector, which can identify patterns in the input^7^. The purpose of the current study was to develop a deep residual learning algorithm to screen for glaucoma from fundus photographs, and to validate its diagnostic performance using an independent dataset.

A recent study suggested the usefulness of applying a deep learning method to diagnose glaucoma^8,9^, however, it used a simple convolutional neural network (CNN), whereas more powerful deep learning methods, such as the Deep Residual Learning for Image Recognition (ResNet)^10^, have now become available. Furthermore, the diagnostic performance of any automated algorithm should be investigated in both highly myopic eyes and non-highly myopic eyes. Previous epidemiological studies have reported that myopia is a risk factor for the development of open angle glaucoma^11–14^. In highly myopic eyes, optic discs are morphologically different from those of non-highly myopic eyes. For instance, tilting of the ONH and thinning of the RNFL is associated with myopia^15,16^. These changes make the detection of glaucoma a challenging task in myopic patients. This is especially important in patients of Asian origin, including Japanese, because myopia is more common in these populations^17,18^. Furthermore, the usefulness of intraocular pressure to detect glaucoma in Asian patients is limited because of the very high prevalence of normal tension glaucoma^19–21^. Thus, a secondary objective of the current study was to validate the diagnostic ability of the deep residual learning algorithm in highly myopic eyes. The potential impact of the deep residual learning algorithm for screening, and therefore the early detection of glaucoma and prevention of blindness, cannot be overstated; fundus photography is commonly used at non-ophthalmological facilities, such as opticians, screening centers and internal medicine clinics.

## Method

The study was approved by the Research Ethics Committee of the Matsue Red Cross Hospital and the Faculty of Medicine at the University of Tokyo. The ethics committee of Matsue Red Cross Hospital waived the requirement for the patient’s informed consent regarding the use of their medical record data in accordance with the regulations of Japanese Guidelines for Epidemiologic Study issued by the Japanese Government, and instead, the protocol was posted at the outpatient clinic to notify participants about the research. This study was performed according to the tenets of the Declaration of Helsinki.

### Subjects

#### Training dataset

The training dataset was prepared using color fundus photographs recorded with a fundus camera (nonmyd WX, Kowa Company, Ltd., Aichi, Japan) between the period of February 2016 and October 2016 at Matsue Red Cross Hospital. All photographs were taken with an angle of view of 45° and resolution of 2144 pixels × 1424 pixels. These photographs were recorded as JPEG images. In total, about 16,000 photographs were reviewed by a single ophthalmologist and glaucoma specialist (M.T.) and 1,364 photographs were assigned a fundus with glaucomatous appearances classification (glaucomatous dataset) and 1,768 photographs were assigned a fundus without glaucomatous appearances classification (normative dataset). For photograph selection, photographs with defocused, un-cleared, too dark, too bright, decentered from the posterior pole, other conditions that could interfere with a diagnosis of glaucoma, or duplication were excluded. Photographs from two eyes of a patient were included, if both photographs satisfied the criteria. Labeling of glaucoma was performed according to the recommendations of the Japan Glaucoma Society Guidelines for Glaucoma^22^; signs of glaucomatous changes were judged comprehensively, such as focal rim notching or generalized rim thinning, large cup-to-disc ratio with cup excavation with/without laminar dot sign, retinal nerve fiber layer defects with edges at the optic nerve head margin, disc edge hemorrhages, and peripapillary atrophy. Other optic nerve head pathologies such as optic nerve/optic nerve head hypoplasia and optic nerve pit, and other retinal pathologies such as retinal detachment, age-related macular degeneration, myopic macular degeneration, macular hole, diabetic retinopathy, arterial and venous obstruction were carefully excluded, but mild epiretinal membrane (without any apparent retinal traction) and mild drusen (without any apparent degeneration) were not excluded. Fundus photographs free of signs of glaucoma and other optic nerve head/retinal pathologies were assigned to the normative dataset. In preparation of the training dataset, subjects’ demographics, such as age and sex, and other ophthalmological findings such as visual field defects, intraocular pressure level, gonioscopic appearance, and OCT measurements were not considered in the diagnosis of glaucoma.

#### Testing dataset

To prepare the testing dataset, a database of spectral-domain OCT (RS-3000 Advance, Nidek, Gamagori, Japan) images was filtered to an examination period from September 2016 to September 2017. The testing dataset consisted of (i) 33 eyes of 33 non-highly myopic glaucoma patients (‘G’ group), (ii) 28 eyes of 28 highly myopic glaucoma patients (‘mG’ group), (iii) 27 eyes of 27 non-highly myopic normative subjects (‘N’ group) and (iv) 22 eyes of 22 highly myopic normative subjects (‘mN’ group). The optic nerve head and macula were scanned using the RS-3000 OCT in the glaucoma mode. Best-corrected visual acuity (BCVA) was measured with a decimal visual acuity chart and then converted into logarithm of the mimimum angle of resolution (logMAR). Axial length was measured with the OA-1000 optical biometer (Tomey, Nagoya, Japan), refractive error was recorded using the RC-5000 refract-keratometer (Tomey) and IOP was determined using the RC-5000 non-contact tonometer (Tomey) or Goldmann applanation tonometer. Posterior fundus photographs were captured using the Kowa nonmyd WX camera. The ‘G’ group was defined as having a spherical equivalent refractive error (SERE) of larger than -6D, glaucomatous changes in fundus photographs, and corresponding thinning in circumpapillary retinal nerve fiber layer thickness (cpRNFLT) measurements and/or in macular inner retinal thickness (mIRT) measurements (outside of the OCT’s normal database), and no other optic nerve/optic nerve head and retinal pathologies by fundus photographs and OCT images. The ‘mG’ group was defined as having a SERE of -6D or smaller, otherwise the same glaucomatous criteria were applied as those for the ‘G’ group. The ‘N’ group was defined as having a SERE larger than -6D and being free of glaucomatous changes and retinal pathologies in both fundus photographs and OCT images. The ‘mN’ group was defined as having a SERE of -6D or smaller and being free of glaucomatous changes and retinal pathologies in both fundus photographs and OCT images. cpRNFLT at the 3.45 mm diameter and vertical cup-to-disc (vC/D) ratio in the raster scanning over a 6 × 6-mm^2^ area centered on the optic disc, and mIRT within the 9-mm circle in the raster scanning over a 9 × 9-mm^2^ area centered on the foveal center were retrospectively obtained. Thus, the testing dataset was prepared without considering VF defects, IOP level, and gonioscopic appearance. A diagnosis of glaucoma was then independently judged by three ophthalmologists specializing in glaucoma (M.T., H.M., and R.A.), photographs were excluded if the diagnoses of the three examiners did not agree.

#### Deep Residual Learning for Image Recognition (ResNet)

The ResNet^10^ is an enhanced deep learning algorithm based on a CNN. Deep training networks enable the extraction of more complex and detailed features from images, however, a deep CNN often suffers from low diagnostic performance due to the vanishing gradient problem and the gradient divergence problem, which hampers the transmission of information from shallow layers to deep layers. In contrast, ResNet avoids these issues by using ‘identity shortcut connections’ that skip one or more layers. Prior to the application of ResNet, fundus images were trimmed around the optic disc (64 × 64 pixels) using the Hough Transform technique^23^, and only this image was inputted into ResNet. The input data in the training dataset were augmented by inverting the fundus images horizontally and vertically, which is useful to prevent overfitting. Luminance values of the fundus images were altered, also for the purpose of preventing overfitting (luminance values between 0 and 100 were transformed to 0, between 100 and 190: linear mapping, and between 190 and 255: 255). The ResNet output is a numerical likelihood of glaucoma for each photograph.

#### Diagnosis by Residents in Ophthalmology

The fundus photographs of the testing dataset were reviewed by Residents in Ophthalmology (A: first year in Ophthalmology residency, B: third year in Ophthalmology residency, C: fourth year in Ophthalmology residency). All of the fundus photographs were reviewed, masking other clinical information. Each Resident made a diagnosis independent to other Residents.

#### Statistical analysis

Three-fold cross validation was performed; the training dataset was divided into three equally sized subsets and the deep learning algorithm was trained using two of the three arms, diagnostic accuracy was then calculated in the remaining arm. The process was iterated three times so that each of the three arms was used as a validation dataset once.

Independent validation was carried out using the testing dataset. The deep learning algorithm was built using all data in the training dataset and the area under the receiver operating characteristic curve (AROC) was calculated. AROCs were also calculated separately for the non-highly myopic and highly myopic eyes (i.e., between the G and N groups and between the mG and mN groups). Sensitivity was calculated at specificity equal to 95% in all of the analyses. AROCs were also obtained based on the diagnoses of glaucoma from the three Residents in Ophthalmology.

As a further comparison, AROC values were calculated using: (i) a CNN with 16 layers, similar to VGG16^24^, (ii) a support vector machine^25^ and (iii) a Random Forest^26^. The details of each method follow;

i) CNN with 16 layers, similar to VGG16.
ii) Support vector machine: Radial Basis Function, Penalty parameter = 1.0.
iii) Random Forest: number of trees = 10,000, criterion = Gini index, minimum number of samples required to split an internal node = 2, The minimum number of samples required to be at a leaf node = 1.

All statistical analyses were carried out using the statistical programming language Python (ver. 2.7.9, Python Software Foundation, Beaverton, US). AROCs were compared using DeLong’s method^27^. Benjamini’s method^28^ was used to correct P values for the problem of multiple testing.

## Result

The testing dataset consisted of (i) 33 eyes of 33 non-highly myopic glaucoma patients (G group), (ii) 28 eyes of 28 highly myopic glaucoma patients (mG group), (iii) 27 eyes of 27 non-highly myopic normative subjects (N group) and (iv) 22 eyes of 22 highly myopic normative subjects (mN group). Demographic data of the subjects are summarized in Table 1.

**Table 1:** Demographics of subjects in testing dataset.

n	G	mG	N	mN	p value
	33	28	27	22	
Age (years)					
Mean ± SD	68.7 ± 7.9	60.2 ± 12.1	66.9 ± 10.3	42.1 ± 16.6	<0.0001a
95% CI	65.9–71.5	55.5–64.9	62.8–71.0	34.8–49.5	
Sex					
Men, n (%)	12 (36)	12 (43)	15 (56)	7 (32)	0.3324b
Women, n (%)	21 (64)	16 (57)	12 (44)	15 (68)	
Eye					
Right, n (%)	19 (58)	16 (57)	8 (30)	11 (50)	0.1160b
Left, n (%)	14 (42)	12 (43)	19 (70)	11 (50)	
BCVA (LogMAR)					
Mean ± SD	0.02 ± 0.14	0.08 ± 0.22	−0.03 ± 0.07	−0.05 ± 0.05	0.0049a
95% CI	−0.03–0.06	0.00–0.16	−0.06–0.00	−0.08–−0.03	
IOP (mmHg)					
Mean ± SD	13.5 ± 3.0	15.6 ± 5.5	13.3 ± 2.9	14.7 ± 2.9	0.0751a
range	12.4–14.5	13.5–17.8	12.2–14.5	13.4–16.0	
Spherical equivalent refractive error (D)					
Mean ± SD	−1.7 ± 2.2	−9.1 ± 3.1	−0.45 ± 2.3	−8.3 ± 1.7	<0.0001a
range	−2.5–−1.0	−10.3–−7.9	−1.4–+0.5	−9.1–−7.5	
Axial length (mm)					
Mean ± SD	24.0 ± 1.4	26.6 ± 1.2	23.6 ± 1.4	26.3 ± 1.1	<0.0001a
range	23.5–24.5	26.2–27.1	23.1–24.2	25.8–26.8	
vC/D ratio					
Mean ± SD	0.73 ± 0.15	0.79 ± 0.11	0.53 ± 0.15	0.37 ± 0.12	<0.0001a
range	0.69–0.79	0.74–0.83	0.48–0.59	0.31–0.42	
cpRNFLT (μm)					
Mean ± SD	71.3 ± 17.0	63.6 ± 14.8	98.0 ± 9.1	97.2 ± 10.4	<0.0001a
range	65.2–77.3	57.8–69.3	94.4–101.6	92.6–101.8	
mIRT (μm)					
Mean ± SD	75.0 ± 13.7	67.1 ± 10.4	97.9 ± 7.6	94.4 ± 6.2	<0.0001a
range	70.2–79.9	63.0–71.1	94.9–100.9	91.7–97.2	

P values are calculated among the four groups by one-way analysis of variance (ANOVA) (a) for continuous variables, and by the chi-square test (b) for categorical variables.

G: non-highly myopic glaucoma subjects, mG: highly myopic glaucoma subjects, N: non-highly myopic normative subjects, mN: highly myopic normative subjects, SD: standard deviation, 95% CI: 95% confidence interval, BCVA, best-corrected visu al acuity, logMAR: logarithm of the minimum angle of resolution, IOP: intraocular pressure, D: diopter, vC/D ratio: vertical cup-to-disc ratio, cpRNFLT: circumpapillary retinal nerve fiber layer thickness, mIRT: macular inner retinal thickness.

The AROC values obtained with the internal verification are shown in Table 2. The diagnostic accuracy varied between 94.2 and 96.0%.

**Table 2:** AROC values obtained with internal three-fold cross validation.

	Fold 1 for validation	Fold 2 for validation	Fold 3 for validation
Iteration 1 (%)	95.0	94.1	96.0
Iteration 2 (%)	94.2	94.9	95.9
Iteration 3 (%)	95.2	94.6	95.3

AROC values obtained were obtained using three-fold cross validation.

**Figure 1:**
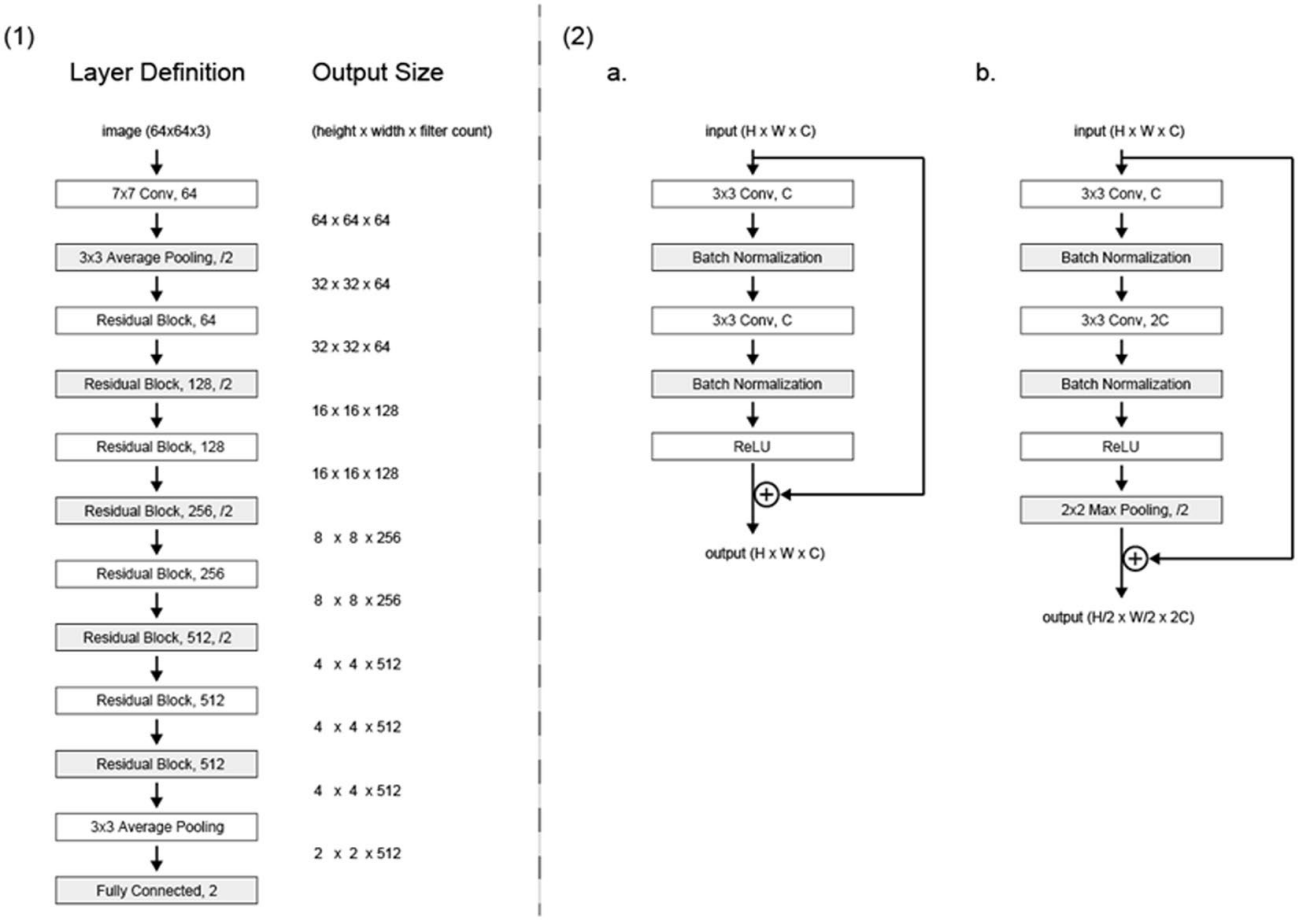
The deep residual learning algorithm to diagnose glaucoma using fundus photography. In ResNet, after two instances of convolution and batch normalization, the input is added to the raw output. (**a**) Shows the scheme of the classifier. The network is highly influenced by ResNet, which has skipping connections in each residual block to promote efficient training of deeper layers. This network has 18 convolutional layers in total. (**b**) shows the detailed explanation of residual blocks. In the case of (**a**), the shape of input and output will be the same. On the other hand, (**b**) doubles the number of channels with the second convolution, while width and height are halved with max pooling. When adding the input to output, half of the filters added are zero-padded so that the shapes match. ResNet: residual network.

**Figure 2:**
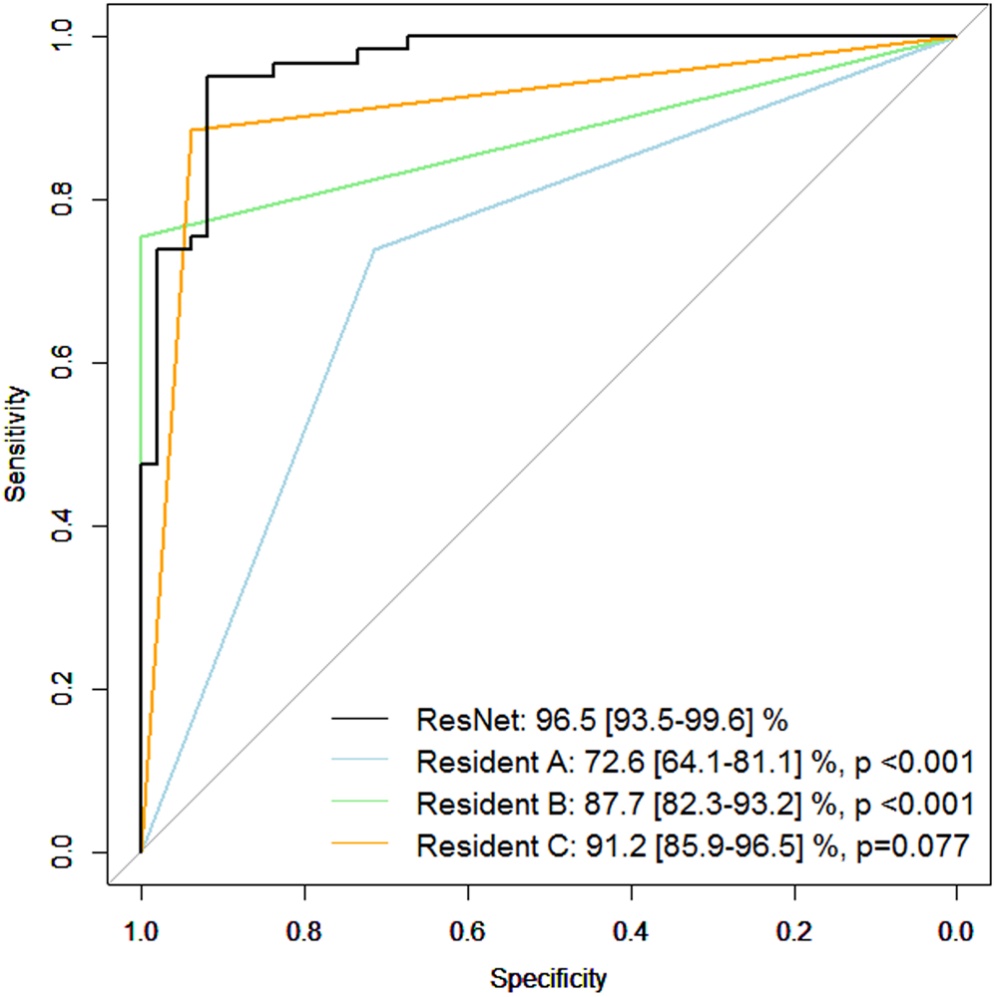
External validation: Receiver operating characteristic curve. The receiver operating characteristic curve obtained in the testing dataset (N = 110). The AROC with ResNet was 96.52 (95% confidence interval [CI]: 95.6 to 98.7)%. The AROC values of the three Residents in Ophthalmology were: 72.6 (95% CI: 64.1 to 81.1)%, 87.7 (95% CI: 82.3 to 93.2)%, and 91.2 (95% CI: 85.9 to 96.47)%, which were significantly smaller than that of ResNet. P values were obtained by comparing the AUC with ResNet and those of Residents in Ophthalmology A, B, C (DeLong’s method with adjustment for multiple comparisons). AROC: area under the receiver operating characteristic curve. ResNet: residual network.

Figure 1 shows the structure of the deep learning algorithm (ResNet) with various parameters (Table 3). Figure 2 shows the receiver operating characteristic curve obtained with all data in the testing dataset. The AROC with ResNet was 96.5 (95% confidence interval [CI]: 93.5 to 99.6)%. The AROCs obtained from the three Residents in Ophthalmology are also shown; their AROCs were A: 72.6 (95% CI: 64.1 to 81.1), B: 87.7 (95% CI: 82.3 to 93.2) and C: 91.2 (95% CI: 85.9 to 96.5)%. The AROC with ResNet was significantly larger than the AROCs of the Residents in Ophthalmology A and B (both p < 0.001, DeLong’s method with adjustment for multiple comparisons). There was not a significant difference between the AROC of ResNet and the AROC of the Resident in Ophthalmology C (p = 0.077).

**Table 3:** Parameters used in ResNet.

Learning Rate	Dropout	Batch Size	Momentum SGD	
			Damping coefficient	Weight Decay
05 to 0.1	0.5	64	0.9	0.0001

SGD: stochastic gradient descent, ResNet: residual network. Learning Rate exponentially decayed as training progressed.

**Figure 3:**
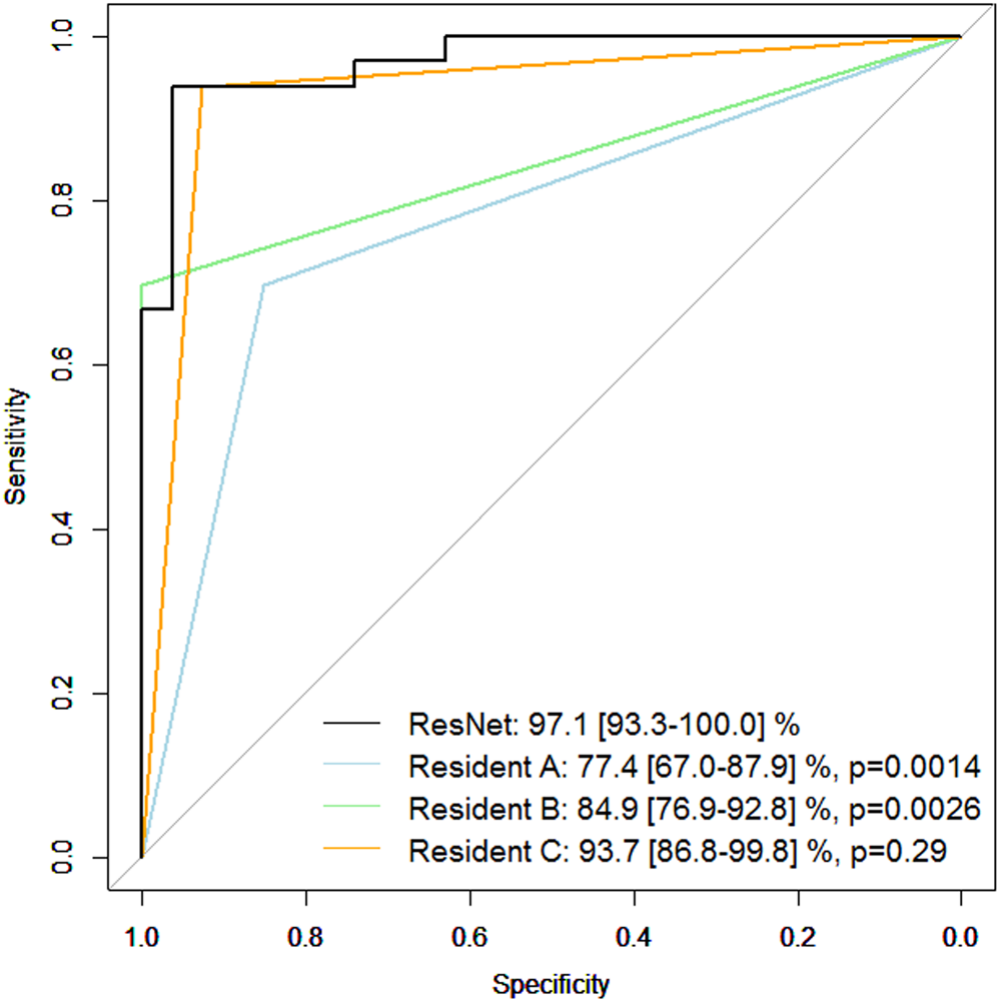
Receiver operating characteristic curve obtained between G and N groups in testing dataset. The receiver operating characteristic curve obtained between G and N groups in the testing dataset. The AROC with ResNet was 97.1 (95% confidence interval [CI]: 93.3 to 100.0)%. The AROC values of the three Residents in Ophthalmology were: 77.4 (95% CI: 67.0 to 87.9)%, 84.9 (95% CI: 76.9 to 92.8)%, and 93.7 (95% CI: 86.8 to 99.8)%. P values were obtained by comparing the AUC with ResNet and those of Residents in Ophthalmology A, B, C (DeLong’s method with adjustment for multiple comparisons). AROC: area under the receiver operating characteristic curve. ResNet: residual network, G: non-highly myopic glaucoma patients and N: non-highly myopic normative subjects.

Figure 3 shows the receiver operating characteristic curve obtained with the G and N groups in the testing dataset. The AROC with ResNet was 97.1 (95% confidence interval [CI]: 93.3 to 100.0)%. The AROCs obtained from the three Residents in Ophthalmology are also shown; their AROCs were A: 77.4 (95% CI: 67.0 to 87.9) and B: 84.9 (95% CI: 76.9 to 92.8)% (p values: < 0.001 and 0.0013, respectively), but not with C: 93.7 (95% CI: 86.8 to 99.8)% (p = 0.15). The AROC with ResNet was significantly larger than the AROCs of the Residents in Ophthalmology A and B (p = 0.0014 and 0.0026, respectively). There was not a significant difference between the AROC of ResNet and the AROC of the Resident in Ophthalmology C (p = 0.29).

**Figure 4:**
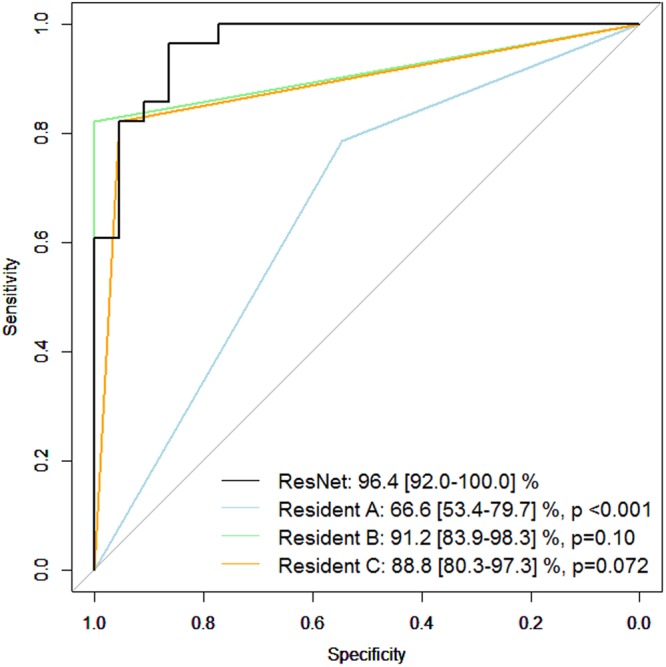
Receiver operating characteristic curve obtained between mG and mN groups in testing dataset. The receiver operating characteristic curve was obtained between mG and mN groups in the testing dataset. The AROC with ResNet was 96.4 (95% CI: 92.0 to 100.0)%. The AROC values of the three Residents in Ophthalmology were 66.6 (95% CI: 53.4 to 79.9)%, 91.2 (95% CI: 83.9 to 98.3)%, and 88.8 (95% CI: 80.3 to 97.3)%. P values were obtained by comparing the AUC with ResNet and those of Residents in Ophthalmology A, B, C (DeLong’s method with adjustment for multiple comparisons). AROC: area under the receiver operating characteristic curve. ResNet: residual network. mG: highly myopic glaucoma patients and N: highly myopic normative subjects.

Figure 4 shows the receiver operating characteristic curve obtained with the mG and mN groups in the testing dataset. The AROC with ResNet was 96.4 (95% CI: 92.0 to 100.0)%. The AROCs obtained from the three Residents in Ophthalmology are also shown; A: 66.6 (95% CI: 53.4 to 79.7), B: 91.2 (95% CI: 83.9 to 98.3), C: 88.8 (95% CI: 80.3 to 97.3)%. The AROC with ResNet was significantly larger than the AROC of the Resident in Ophthalmology A (p < 0.001). There was not a significant difference between the AROC with ResNet and the AROCs of the Residents in Ophthalmology B and C (p = 0.10 and 0.072, respectively).

The AROC values associated with the other algorithms are shown in Table 4; these varied between 66.6 (95% CI: 53.4 to 79.7)% with the Support Vector Machine and 91.2 (95% CI: 83.5 to 99.0)% with a CNN with 16 layers, similar to VGG16.

**Table 4:** AROC values obtained with other models used to diagnose glaucoma.

	all eyes (N = 110)	N and G groups	mN and mG groups
a CNN with 16 layers, similar to VGG16	86.3 [79.9–93.0]	81.8 [71.2–91.4]	91.2 [83.5–99.0]
Random Forests	77.5 [69.6–85.4]	76.8 [65.9–87.7]	78.3 [66.9–89.6]
Support Vector Machine	71.1 [62.7–79.5]	75.1 [64.1–86.1]	66.2 [53.0–79.5]

AROC [95% confidence interval] values were calculated by training using (i) CNN with 16 layers, similarly to VGG16, (ii) support vector machine, and (iii) Random Forest, using all of the training dataset, and validating using the testing dataset.

AROC: area under the receiver operating characteristic curve, CNN: convolutional neural network, G: non-highly myopic glaucoma patients, N: non-highly myopic normative subjects, mG: highly myopic glaucoma patients and N: highly myopic normative subjects.

## Discussion

A deep residual learning algorithm to screen for glaucoma from fundus photographs was developed using a training dataset that consisted of 1,364 eyes with open angle glaucoma and 1,768 eyes of normative subjects. The diagnostic performance of this algorithm was validated using independent testing datasets. The AROC of the deep residual learning algorithm was 96.5% with all eyes, 97.1% between the G and N groups, and 96.4% between the mG and mN groups. These AROC values tended to be significantly larger than those from Residents in Ophthalmology. We also investigated the diagnostic performance of other deep learning models and machine learning methods, however, these algorithms resulted in much lower AROC values (see Table 4). As a scientific merit, the current results have shown the modern powerful deep learning method of the ResNet^10^ enabled an accurate diagnosis of glaucoma from fundus photographs. Diagnosing glaucoma in highly myopic eyes is a challenging task, because of the morphological difference from those of non-highly myopic eyes^15,16^, however the current results suggested the constructed algorithm had a high diagnostic power in such eyes.

Deep learning methods to diagnose disease from fundus photographs have been reported previously. Gulshan *et al*. developed a CNN, trained with 128,175 fundus photographs, to detect diabetic retinopathy^29^. The AROC of this algorithm was 99%. Takahashi *et al*. applied the GoogLeNet model to the same diagnostic problem and achieved 81% accuracy^30^. The task of glaucoma detection may be more challenging than the diagnosis of diabetic retinopathy, since diagnosis of diabetic retinopathy is based on abnormal retinal features, such as hemorrhage, microaneurysm and exudates, whereas the diagnosis of glaucoma relies on the estimation of subtle changes in the shape of the optic disc. Such alterations are better assessed using stereo-photographs, but two-dimensional fundus photographs were used in the current study, making the task even more challenging. Nonetheless, the deep residual learning model achieved very good discrimination with an AROC between 96.4 and 97.1%. It is difficult to directly compare the diagnostic performance of the current deep learning algorithm with those in recent studies^8,9^ since the diagnosis of glaucoma depends on many factors, including the stage of glaucoma and refractive status of the eye. In the current study, the ResNet algorithm was trained using a much smaller number of eyes (1,364 color fundus photographs with glaucomatous indications and 1,768 color fundus photographs without glaucomatous features) than in previous studies (approximately 120,000 and 40,000 fundus photographs), however, its diagnostic performance was equal to, or superior to, Residents in Ophthalmology, both in non-highly myopic and highly myopic eyes. It should be noted that high myopia was the top reason for false negative classification in^8^, despite the large size of the training dataset (approximately 40,000 fundus photographs). In contrast, our results suggested an accurate diagnosis can be obtained in highly myopic eyes.

The merits of applying machine learning methods to diagnose glaucoma have been widely reported. We previously applied the Random Forests method to diagnose glaucoma based on OCT measurements. As a result, the AROC to discriminate glaucomatous (from early to advanced glaucoma cases) from normative eyes was 98.5%^31^. We also reported that the AROC of this approach was 93.0% when discriminating early stage glaucoma patients and normative eyes^32^. Following great successes of deep learning methods for discrimination tasks in various fields, the application of these methods have just begun in the field of glaucoma. Indeed we have very recently reported the merit of applying deep learning methods to predict visual field sensitivity from OCT^33^. Although a more detailed investigation of glaucomatous retinal damage can be made using OCT, compared to fundus photography, the potential impact of the current algorithm as a screening tool cannot be exaggerated; fundus photography is commonly used at screening centers, opticians and internal medicine clinics.

The ResNet deep network (18 convolutional layers were used in the current study) enables the extraction of complex and detailed features from images. To avoid the model identifying features from other parts of the retina, outside the optic disc, images were cropped before training the model. This also reduced the model learning duration time. However, other glaucomatous findings might be observed outside the optic disc on fundus photographs, such as nerve fiber layer defects and optic disc hemorrhage. A future study should investigate whether considering this information improves diagnostic accuracy. Furthermore, it is possible that a deep learning algorithm trained on stereoscopic fundus photographs or OCT images offers better discrimination than the one built here on two-dimensional fundus photographs. The principal purpose of the current study was to build an automated screening tool for glaucoma with high discriminatory power, which could be used in the majority of screening facilities. The significant disadvantage of a screening tool based on stereoscopic fundus photography or OCT is that its use would be very limited since these technologies are equipped in only a limited number of ophthalmological facilities, while two-dimensional fundus photography is much more widely available. Nonetheless, this does not deny the value of a screening tool based on OCT or stereoscopic fundus photography, and a future study should investigate this possibility. A limitation of the current study concerns photograph selection, photographs with features that could interfere with an expert diagnosis of glaucoma were omitted. Excluding these images means further testing of the algorithm is essential to measure its performance as a screening tool in a “real world” setting. However, identifying low quality images is usually much easier than automatically screening for glaucoma.

In conclusion, a deep residual learning algorithm was developed to automatically screen for glaucoma in fundus photographs. The algorithm had a high diagnostic ability in non-highly myopic and highly myopic eyes.
